# Washington State’s Fruit and Vegetable Prescription Program: Improving Affordability of Healthy Foods for Low-Income Patients

**DOI:** 10.5888/pcd16.180617

**Published:** 2019-07-18

**Authors:** Jessica Marcinkevage, Alyssa Auvinen, Susmitha Nambuthiri

**Affiliations:** 1 Prevention and Community Health, Washington State Department of Health, Olympia, Washington

## Abstract

**Purpose and Objectives:**

We conducted a mixed-methods process and outcome evaluation for a statewide fruit and vegetable prescription program. The process evaluation assessed program implementation, identified opportunities for quality improvement, and provided recommendations for future programmatic activities. The outcome evaluation measured how the program affected purchases of fruits and vegetables among low-income patients and patient satisfaction with the program.

**Intervention Approach:**

The Washington State Department of Health (WA DOH) partnered with public and private health care systems, public health agencies, a community-based organization, and a supermarket chain to launch a fruit and vegetable prescription program in 2016. The prescription was a $10 voucher redeemable for fruits and vegetables at any one of 169 participating supermarkets. Prescriptions were distributed to eligible low-income patients in clinics and community settings.

**Evaluation Methods:**

WA DOH reviewed quarterly reports, meeting minutes and notes, telephone call logs, and email logs to solicit feedback on program implementation processes. We calculated overall prescription redemption rates on the basis of the number of prescriptions distributed by implementing partners and the number of prescriptions redeemed at participating supermarkets. We assessed patient satisfaction through a web-based survey. The study period was July 1, 2016, through June 30, 2018.

**Results:**

Best practices for implementation included using the prescription to improve patient engagement and retention and connect patients to additional services, and working in the community to enhance program support and uptake. Overall, $154,810 in fruit and vegetable prescriptions were redeemed during the study period (54.4% redemption rate). Most survey respondents (88.2%) reported eating more fruits and vegetables than previously as a result of the prescription.

**Implications for Public Health:**

Fruit and vegetable prescriptions are an effective way to increase affordability of healthy foods for low-income patients. These programs are scalable and translatable across various types of patient–provider encounters.

**Summary**

**What is already known on this topic?**

Programs that increase affordability of fruits and vegetables through financial incentives have improved fruit and vegetable consumption and food security among participants. However, program scalability is limited when programs rely on partnerships with farmers markets and small-scale grocers.

**What is added by this report?**

Through a process and outcome evaluation, this report highlights program implementation successes and barriers of providing fruit and vegetable prescriptions in partnership with a supermarket chain to low-income residents in Washington State.

**What are the implications for public health practice?**

A statewide fruit and vegetable prescription program is scalable and translatable across various types of patient–provider encounters and helps improve affordability of fruits and vegetables for low-income residents.

## Introduction

Despite public health efforts, people in Washington State and the United States overall do not eat enough fruits and vegetables to meet national recommendations for a healthy diet (1–3). This is especially true for people who have limited access to healthy foods (4,5). Food insecurity — the limited or uncertain availability of nutritionally adequate and safe foods (6) — disproportionately affects people with low incomes, people of color, and rural residents (7). Food insecurity has a negative effect on health and increases the risk of developing chronic diseases such as type 2 diabetes (8–10) and hypertension (8,11). Increased fruit and vegetable consumption can mitigate the progression of chronic disease (12,13) and is associated with reduced risk of cardiovascular disease (14,15), cancer (16), stroke (14,17,18), and premature death (17,19).

Although in 2017 the overall food insecurity rate in Washington State (11.5%) was lower than the national average (12.5%), intrastate geographic and demographic disparities exist; the rate in some Washington counties is nearly 1.5 times the national average (20). Federal nutrition assistance programs, such as the Supplemental Nutrition Assistance Program (SNAP), are effective in significantly reducing food insecurity (21). However, SNAP participants are less likely than income-eligible and higher-income nonparticipants to consume fruits and vegetables (22,23). Programs that increase affordability of fruits and vegetables through financial incentives have improved rates of fruit and vegetable consumption (24) and food security (25) among participants overall and improved glycemic control among participants with diabetes (26). However, such programs are limited when they rely on partnerships with farmers markets that may operate only seasonally, or small-scale grocers that may carry only a small variety of fruits and vegetables. 

The US Department of Agriculture’s Food Insecurity Nutrition Incentive (FINI) Grant Program supports projects that incentivize the purchase of fruits and vegetables among SNAP participants (27). In 2015, the Washington State Department of Health (WA DOH) received a FINI grant to improve the nutritional quality of SNAP participants’ diets in Washington State by implementing fruit and vegetable incentive programs with food retailers and community partners (www.doh.wa.gov/FINI). As part of the FINI grant, WA DOH began implementing a statewide fruit and vegetable prescription program in July 2016.

## Purpose and Objectives

The objective of this study was to describe mixed-method process and outcome evaluation results after 2 years of implementation of the fruit and vegetable prescription program, using data collected from July 1, 2016, through June 30, 2018 (hereinafter, “the study period”). The purpose of the process evaluation was to 1) examine strengths and weaknesses of the fruit and vegetable prescription program implementation and 2) gain insight into successful programming activities for fruit and vegetable prescriptions. The purpose of the outcome evaluation was to 1) assess overall effectiveness of the program in improving affordability of healthy foods among low-income patients and 2) assess patient satisfaction with the fruit and vegetable prescription program. Although the program is planned to run through December 2019, the reporting of mid-program evaluation findings, given the current national climate for fruit and vegetable incentives (28), can help other health departments and interested parties in implementing similar programs.

## Intervention Approach

**Figure 1:**
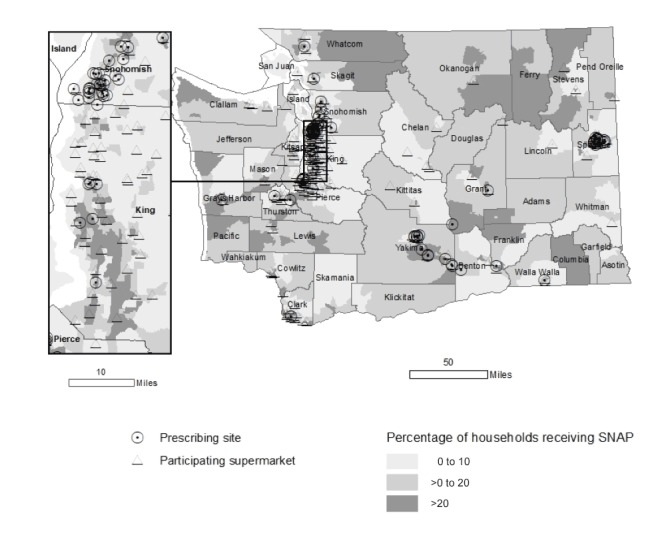
Percentage of households receiving Supplemental Nutrition Assistance Program benefits by census tract and location of prescribing sites and participating supermarkets, Washington State’s Fruit and Vegetable Prescription Program, 2016–2018. Resources: Supplemental Nutrition Assistance Program data, American Community Survey, 2012–2016; clinic data, Healthy Eating Active Living Program, Washington State Department of Health.

In July 2016, WA DOH partnered with public and private health care systems, public health agencies, and a community-based organization (hereinafter, “implementing partners”), and a supermarket chain to launch a fruit and vegetable prescription program in counties where the prevalence of low fruit and vegetable intake, food insecurity, and chronic disease are disproportionately high (29). The fruit and vegetable prescription is a $10 fruit and vegetable voucher redeemable at any one of 169 participating supermarkets — defined as a store containing all major food departments and reporting at least $2 million in annual sales (30) — belonging to the supermarket chain (Figure 1). WA DOH designed the program on the basis of a 2014 fruit and vegetable prescription pilot program in Washington State with one participating health care system and the supermarket chain, and in consideration of the modeled health effects of fruit and vegetable incentives (31).

Programmatic planning exercises during the first 15 months of the grant period (April 2015–June 2016) included an in-person meeting, email correspondence, and webinars with implementing partners. Through these exercises, WA DOH and implementing partners identified that they needed flexibility in implementing the fruit and vegetable prescription program.

Implementing partners used various types of patient encounters, prescribers, and dosing amounts (Table), which were determined by the needs of their diverse patient populations — including racial/ethnic minority groups, senior citizens, and residents in urban and rural areas — and typical workflows. The prescription — available in English, Spanish, and Russian — was distributed to patients during one-on-one clinic visits; disease prevention and management classes (including the Diabetes Prevention Program [32] and Chronic Disease Self-Management Program [33]); maternal, infant, and child health visits; community events; health education classes; and nutrition education classes. Prescribers hand-wrote a 1-month expiration date on the prescription at the time of distribution. Patients were required to be a SNAP participant to be eligible for the program, and prescribers confirmed eligibility verbally or through a questionnaire. The number of prescriptions (ie, “dose”) received by patients varied across implementing partners, encounter types, and frequency of encounters (Table). We established no limit on the number of times a patient could receive a prescription (eg, in some settings patients received a prescription once per week for 6 months), and patients could receive prescriptions from more than one implementing partner. In some settings, adults received the prescription on behalf of children younger than 18 years. Implementing partners tracked prescription distribution via paper method or electronic medical record (EMR). Once per quarter, implementing partners reported monthly distribution numbers to WA DOH through an electronic data collection system. One or more unique Price Look Up (PLU) numbers was assigned to each implementing partner and was printed on the prescription.

**Table:** Summary of Fruit and Vegetable Prescription Program Implementation Characteristics Across Implementing Partners, Washington State, 2016–2018

Partner ID No.	Implementing Partner type	Patient Encounter Type	Prescribers	Dose	Patient Population^a^	Distribution Period
1	Federally qualified health center	• Maternal, infant, and child health visits^b^ • Nutrition education classes	Dietitians/nutritionists (n = 4), nurses (n = 4), social workers (n = 9), health educators (n = 2)	Varied according to family size and encounter frequency	Adults and children	July 2016–June 2018
2	Federally qualified health center	• Clinic visits • Health education classes	Health educators (n = 12)	1 or 2 Prescriptions per week, depending on family size	Adults	January 2018–June 2018
3	Federally qualified health center	• Clinic visits • Disease management and/or prevention classes^c^ • Maternal, infant, and child health visits^b^ • Nutrition education classes	Dietitians/nutritionists (n = 9), nurses (n = 6), social workers (n = 3), health educators (n = 9), community health workers (n = 8)	Varied according to family size and encounter frequency	Adults and children	April 2018–June 2018
4	General hospital	• Disease management and/or prevention classes^c^ • Maternal, infant, and child health visits^b^	Dietitians/nutritionists (n = 4), nurses (n = 12), health educators (n = 11)	Varied according to family size and encounter frequency	Adults and children	July 2016–June 2018
5	General hospital	• Community events • Maternal, infant, and child health visits^b^ • Nutrition education classes	Dietitians/nutritionists (n = 2), social workers (n = 2) clinician (n = 1), health educators (n = 1), outreach workers^d^ (n = 7)	1 Prescription per encounter	Adults	July 2016–June 2018
6	Pediatric primary care clinic	• Clinic visits	Social workers (n = 2)	1 or 2 Prescriptions per week, depending on family size	Children	July 2016–June 2018
7	Outpatient medical clinics	• Clinic visits	Dietitians/nutritionists (n = 5)	2 Prescriptions per week	Adults	May 2018–June 2018
8	Public hospital district^e^	• Clinic visits • Community events • Nutrition education classes • Health education classes	Dietitians/nutritionists (n = 8), social workers (n = 2), outreach workers^d^ (n = 14)	Varied according to family size and encounter frequency	Adults and children	March 2018–June 2018
9	Tribal health department	• Clinic visits • Community events • Nutrition education classes	Dietitians/nutritionists (n = 1), nurses (n = 1), social workers (n = 1), outreach workers^d^ (n = 2)	1–4 Prescriptions per encounter, depending on family size	Adults and children	April 2018–June 2018
10	Local health department	• Community events • Maternal, infant, and child health visits^b^ • Nutrition education classes	Dietitians/nutritionists (n = 3), health educators (n = 15), community health workers (n = 17)	1 Prescription per encounter	Adults and children	July 2016–June 2018
11	Local health department	• Community events • Nutrition education classes	Health educator (n = 1), community health workers (n = 2)	1 Prescription per encounter	Adults	July 2016–June 2018
12	Local health department	• Nutrition education classes	Health educators (n = 2)	1 Prescription per encounter	Adults	July 2016–March 2018
13	Local health department	• Community events • Nutrition education classes	Outreach workers^d^ (n = 2)	1 Prescription per encounter	Adults	July 2016–September 2017
14	Community-based organization	• Health education classes	Health educator (n = 1)	1 Prescription per encounter	Adults	July 2016–May 2017

a For all patient populations that include children, adults receive prescriptions on behalf of their children.

b Maternal, infant, and child health visits defined as home visiting, parenting classes, pregnant and postpartum visits, or Special Supplemental Nutrition Program for Women, Infants, and Children (WIC).

c Disease management and/or prevention programs defined as childhood obesity prevention programs, Chronic Disease Self-Management Program (34) or Diabetes Prevention Program (33).

d Outreach workers defined as community-based staff who link patients to health services.

e Public hospital district defined as a governmental entity authorized by Washington State law to deliver health services, including acute hospital care and preventive care.

Patients redeemed the prescription at any one of 169 participating supermarkets in Washington State. Patients presented the prescription at point-of-sale to purchase qualifying items, which included fresh, canned, or frozen fruits and vegetables without added fats, oils, sugars, or salt. No additional purchase was necessary to redeem the prescription, but patients were encouraged to purchase at least $10 of qualifying items per transaction. WA DOH and the supermarket chain provided training to store staff members to prepare for prescription redemption. At the point-of-sale, the prescription was scanned and purchase information was stored in the supermarket’s sales database. The supermarket chain provided data on the number of prescriptions redeemed by PLU, by quantity and characteristics of items purchased, and by dollar amount.

Overall, 14 implementing partners participated during the study period. The program began with 9 implementing partners; 3 implementing partners discontinued distributing prescriptions in 2017 and 2018 because of staffing limitations. In 2018, 5 new implementing partners began distributing prescriptions, resulting in 11 implementing partners with 185 prescribers in 86 prescribing sites in the program in June 2018 (Figure 1).

## Evaluation Methods

To assess the fruit and vegetable prescription program, we conducted a mixed-methods process and outcome evaluation. The process evaluation assessed program implementation, examining strengths and weaknesses of the program and identifying successful programming activities, to identify opportunities for program quality improvement and provide recommendations for future programmatic activities. The outcome evaluation measured program use: how the fruit and vegetable prescription program affected patients’ purchasing of fruits and vegetables and patient satisfaction with the program. We developed the evaluation plan and questions on the basis of extensive stakeholder input through annual in-person meetings and presentations, quarterly telephone calls and webinars, and frequent email communication with implementing partners. The Washington State Institutional Review Board deemed evaluation activities exempt from review.

### Process evaluation: qualitative data analysis of program implementation

As part of process evaluation activities, we reviewed quarterly reports, meeting minutes and notes, and telephone call and email logs to solicit feedback on program implementation processes. Each quarter, as part of regular reporting required for participation in the program, implementing partners identified key successes in their fruit and vegetable prescription program implementation activities and overall experiences with prescription distribution, including facilitators and barriers to effective implementation. We collected this information electronically through a secure online survey platform. All implementing partners responded to the same open-ended questions on facilitators, barriers, and key program activities. WA DOH staff members reviewed these electronic reports each quarter and tracked responses through a Microsoft Excel 2013 spreadsheet, providing technical assistance as needed for continuous program quality improvement. In all, this review included 89 reports generated during the study period.

WA DOH staff members also kept records of telephone calls and emails from implementing partners requesting technical assistance for program implementation and telephone calls and emails that described the steps taken as a result of this assistance. We matched details from telephone call and email logs to implementing partner reporting to identify and confirm quality improvement measures taken. In all, we reviewed records from 20 telephone calls and emails during the study period.

In addition to required implementing partner reporting and requests for technical assistance, WA DOH staff members held quarterly meetings, including 6 virtual meetings and 2 in-person meetings, during the study period. These meetings were an opportunity for the study team to ground-truth key themes emerging from reporting and provided a venue for more in-depth knowledge sharing among all implementing partners. WA DOH kept detailed agenda and meeting notes from each of these meetings.

Evaluation staff members reviewed all compiled responses from reporting, technical assistance efforts, and meeting notes and closed-coded responses to answer the following questions:

- What are major facilitators and barriers to 1) program implementation in the clinic setting and 2) patients’ use of a fruit and vegetable prescription?
- What are key activities and/or resources considered critical to the successful implementation of an incentive program in the clinic setting?

Evaluation staff analyzed coded responses and identified patterns across responses by using thematic analysis.

Although one-on-one in-depth interviews were originally planned during the study period as part of the process evaluation, staffing limitations led to a change in methodology and approach. Additionally, preliminary review of documents received from implementing partners and sharing of results with key stakeholders showed that information from regular reporting and technical assistance activities was more than sufficient for identifying facilitators and barriers to program implementation and provided more timely information for continuous program quality improvement than would have been possible from interviews.

### Outcome evaluation: quantitative data analysis of program use and patient satisfaction

We calculated overall prescription redemption rates for each implementing partner as a measure of program use. Each quarter, implementing partners reported the number of prescriptions distributed each month via a secure online portal. Each month, WA DOH also received point-of-sale transaction details for each prescription redeemed, including the PLU, dollar amount spent, and characteristics of items purchased, from the supermarket’s sales database via secure file transfer. Fruits and vegetables purchased at the point of redemption were categorized according to type (fresh, frozen, or canned) and whether they were eligible to be purchased with the prescription (ie, contained no added fats, oils, sugars, or salt). We calculated redemption rates by dividing the number of prescriptions redeemed by the number of prescriptions distributed over the specified time period. We reported rates to implementing partners each quarter. We assessed redemption rates by quarter and time of month (first 10 days of month, second 10 days, and third 10 days) to determine how timing of SNAP benefit issuance affected prescription redemption. We compared redemption rates from earlier in the month (ie, the first and second 10 days of the month, when SNAP benefits would have been issued) with the third 10 days to assess whether the fruit and vegetable prescription was helping to stretch participants’ SNAP benefits. We used data on purchases and redemption data to assess program use to answer the following evaluation question: To what extent did patients use the fruit and vegetable prescription?

We surveyed patients to assess their satisfaction with the fruit and vegetable prescription program. Because response rates for telephone and mail-based surveys are declining (34) and because these survey types are relatively labor-intensive to implement, we required a different approach and chose to test a web-based approach. Although one concern about web-based approaches to data collection is its accessibility among low-income or elderly populations, national data show that most low-income households and households with people 65 years or older use a computer or other handheld device for internet access (35). 

Patients could complete the voluntary survey on any electronic device and were eligible to take the survey each time they received a prescription. When the prescription was distributed to an adult on behalf of a child’s participation, the parent or guardian was invited to take the survey on behalf of the child. The survey consisted of 30 questions, including validated questions on demographic and socioeconomic characteristics (36), food insecurity (37), and fruit and vegetable consumption (38). Additional survey questions were provided or adapted from implementing partner feedback and tested among patient populations as applicable. These questions asked about health and shopping behaviors and general satisfaction with the fruit and vegetable prescription program. Analysis of the survey responses helped answer evaluation questions related to patient satisfaction, namely

- To what extent did patients find the fruit and vegetable prescription acceptable to use?
- How does receipt of a fruit and vegetable prescription change patients’ knowledge, attitudes, and practices toward fruit and vegetable consumption, health behaviors, and perceived access to healthy foods?

The survey first became available to patients in September 2017. Each participant received a $3 electronic gift card at each survey completion (maximum 1 per week). We managed and calculated summary statistics (ie, percentages) by using Microsoft Excel 2013.

## Results

### Perspective of implementing partners

Implementing partners identified several key milestones and lessons learned as a result of the prescription program, illuminating potential areas for future program success.

**Offering the fruit and vegetable prescriptions improved patient visits.** Implementing partners consistently reported increased attendance and retention in health care appointments and community-based classes when prescriptions were offered. For example, one implementing partner reported higher-than-average completion rates among patients in the Chronic Disease Self-Management Program as a result of offering the prescription, with 95% of patients completing the 6-week program. Anecdotal evidence also showed that as a result of the fruit and vegetable prescription program, patients scheduled and kept more follow-up appointments with primary care dietitians, and no-show rates for home visiting decreased slightly.

**Providing a method to identify high-need patients helped connect these patients to additional services.** Several implementing partners incorporated food insecurity screening and nutrition wraparound services into their institutional workflows as a result of offering fruit and vegetable prescriptions. One pediatric primary care clinic referred food-insecure families to an outreach organization that helped families determine eligibility for and enroll in SNAP and other food assistance resources. Another implementing partner worked with community health workers who lived and worked in low-income housing sites to distribute prescriptions during nutrition education events. The community health workers were uniquely situated in these low-income housing sites and connected their peers to other health-related screenings and programs that improve food security and other social determinants of health.

**Working in the community enhanced program support and uptake.** Implementing partners reported using several methods to best reach eligible patients in their communities. One such method was having bilingual dietitians, nutrition educators, and other health care providers distribute fruit and vegetable prescriptions. One implementing partner hosted culturally relevant nutrition education classes in Russian and distributed the prescriptions in the Russian-speaking community. Other implementing partners reported efforts to engage with the Spanish-speaking community; however, some patients expressed hesitancy in enrolling in SNAP for fear of negative consequences to their documentation status.

**Eliminating administrative burden helped ease program implementation. **Implementing partners reported difficulty tracking distribution of the paper-based prescriptions for various reasons. First, the prescriptions required a hand-written expiration date, which increased workload on prescribers, as well as time required for distribution, which could affect patients’ perception of the program. Prescribers in many encounters also had to count out each prescription during distribution, which required time and introduced potential human error in the number of prescriptions distributed. Many prescribers distributed prescriptions outside of traditional clinic visits (eg, at community-based nutrition education classes, during community health worker visits), and they used paper tracking sheets to document distribution because they did not have access to an EMR. Although prescribers could have used technology — for example, tablets or smart phones — for tracking purposes, the use of technology could have been perceived as intrusive to patients or as a barrier to prescribers. Finally, in the few locations where the EMR was available, implementing partners found that introduction of an EMR tracking method was cost-prohibitive because of the involvement of outside vendors or information technology staff.

### Program use and patient satisfaction

**Figure 2:**
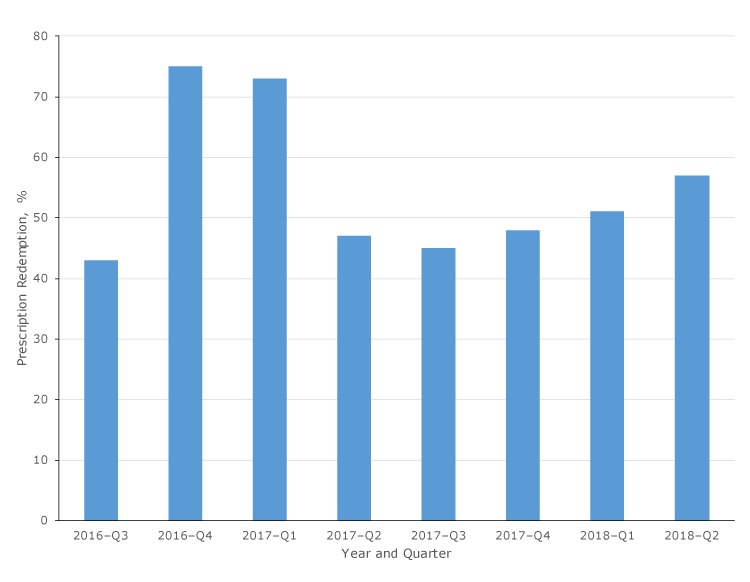
Quarterly redemption rate of prescriptions for all implementing partners combined, Washington State’s Fruit and Vegetable Prescription Program, July 2016–June 2018. Redemption rates were calculated by dividing the number of prescriptions redeemed by the number of prescriptions distributed over the specified time period.

**Redemption rates and other characteristics of fruit and vegetable prescription transactions.** During the study period, 28,481 prescriptions were distributed, with $284,810 provided to patients to use when purchasing fruits and vegetables. Of these, 15,481 prescriptions were redeemed, for an overall redemption rate of 54.4% (15,481 of 28,481). Because each prescription was valid for 1 month from the date of distribution, and the exact date of prescription distribution was not linked with date of redemption, this redemption rate is a conservative estimate; true redemption rates cannot be calculated until one month after the program’s end, in December 2019. Redemption rates varied by quarter (Figure 2), ranging from 42.5% (376 of 884) in the first quarter of operation (July–September 2016) to 72.5% (2,336 of 3,221) in the third quarter of operation (January–March 2017). Rates also varied by implementing partner type, with 2 partners consistently showing redemption rates greater than 50% (partner 1, 64.1% [7,606 of 11,865] and partner 2, 57.1% [3,222 of 5,643]). Among all partners, overall redemption rates measured 33.9% (914 of 2,698) or higher. Redemption rates also varied by time of month. The redemption rate averaged 29.0% (4,489 of 15,481) during the first 10 days of the month, 33.0% (5,109 of 15,481) during the second 10 days, and 38.0% (5,883 of 15,481) during the third 10 days. 

**Table:** 

Year and Quarter	Number of Prescriptions Issued	Number of Prescriptions Redeemed	Prescription Redemption %
2016-Q3	884	376	42.5
2016-Q4	2180	1645	75.5
2017-Q1	3221	2360	73.3
2017-Q2	3252	1582	48.6
2017-Q3	3640	1466	40.3
2017-Q4	3640	1760	48.4
2018-Q1	5164	2618	50.7
2018-Q2	6500	3674	56.5

**Figure 3:**
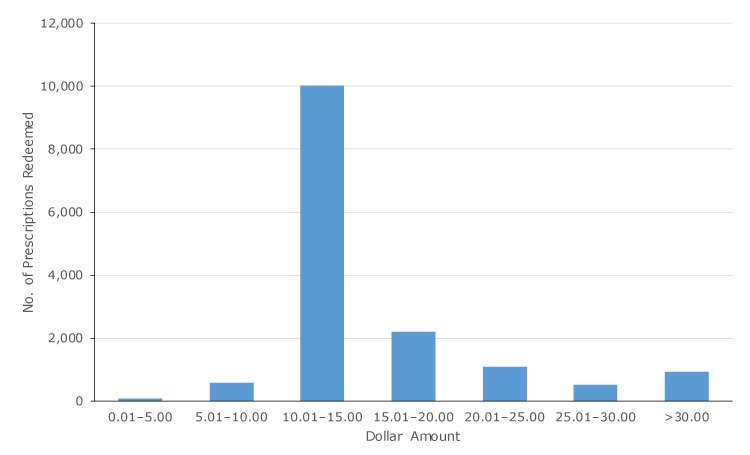
Dollar amount spent on fruit and vegetable purchases per prescription redeemed, Washington State’s Fruit and Vegetable Prescription Program, July 2016–July 2018.

Although we could not track transactions at the patient level, linkage with the supermarket’s loyalty shopper program showed that prescriptions were redeemed by at least 3,688 unique shoppers. In 95.6% of all prescription transactions (14,802 of 15,481), patients spent more than $10.00 on qualifying items (Figure 3). On average, shoppers spent $17.62 (standard deviation, $11.18) on qualifying items during the first shopping trip in which they redeemed a prescription. Although most items (94.0% of dollar amount spent; $145,520 of $154,810) purchased were fresh fruits and vegetables, patients used the prescription for purchase of canned (4.0%; $6,190 of $154,810) and frozen (2.0%; $3,100 of $154,810) fruits and vegetables.

**Table:** 

Dollar Amount	No. of Prescriptions Redeemed
0.01–5.00	91
5.01–10.00	588
10.01–15.00	10,025
15.01–20.00	2,217
20.01–25.00	1,093
25.01–30.00	529
>30.00	938

**Perception of the fruit and vegetable prescription program among patients receiving prescriptions.** From September 1, 2017, through June 30, 2018, 144 patients completed the electronic survey. Most respondents (88.9%; n = 128) reported the prescription was easy to use. Of the 144 respondents, 74.3% (n = 107) reported food in their home was less likely to run out as a result of the prescription, and 86.8% (n = 125) reported increased ability to afford balanced meals.

Patients also reported a perceived increase in fruit and vegetable intake as a result of receiving the fruit and vegetable prescription: 88.2% (n = 127) reported eating more fruits and vegetables than previously and 70.1% (n = 101) reported that they tried a new fruit or vegetable. In addition, 76.4% (n = 110) reported increased fruit and vegetable consumption among family members.

Participation in the program also resulted in patients’ perceived health benefits: 71.5% (n = 103) reported managing their health conditions better, and 81.2% (n = 117) reported improvement in meeting nutrition, diet-related, or meal plan goals.

## Implications for Public Health

Results from the mid-program evaluation affirmed that the fruit and vegetable prescription program improved affordability of fruits and vegetables for low-income patients and helped them achieve their health behavior goals. Our analysis shows patients maximized the full value of the prescription, and stretched limited food budgets to buy healthy foods. Patient-level survey responses showed a perceived improvement in consumption of healthy foods and perceived health benefits as a result of receiving the prescription.

Findings from our evaluation highlight several important points. First, our results show that fruit and vegetable prescription programs are scalable and translatable across various patient–provider encounter types in various geographic settings, but they require flexibility for implementing partners to fit into their typical institutional or programmatic workflows. Despite this requirement of flexibility, offering the prescriptions is an effective way to engage patients in educational and counseling sessions. A key reason for its effectiveness is that the prescription simultaneously addresses food insecurity and chronic disease prevention/management by providing financial support for patients to modify purchases and achieve healthy eating goals.

Second, our evaluation results show how social determinants of health can be incorporated into patient-provider encounters. Implementing partners can capitalize on fruit and vegetable prescription programs by establishing consistent, holistic enrollment criteria and processes for patients. Although funding for this program requires patients to be enrolled in SNAP, a useful screening tool for other fruit and vegetable prescription programs would be the Hunger Vital Sign (37), a validated 2-question food insecurity screening tool that identifies marginally and severely food-insecure patients (39). Screening for food insecurity is preferable to relying on enrollment in nutrition assistance programs to improve program reach, because some patients who are food insecure may not be eligible for or choose to sign up for SNAP benefits for various reasons (eg, income, immigration status). Additionally, patients who screen positive for food insecurity can be referred to other wraparound services such as federal nutrition assistance programs (eg, Special Supplemental Nutrition Program for Women, Infants, and Children [WIC], SNAP, senior nutrition programs) and community resources (eg, food banks). As evidenced by implementing partners’ inclusion of community members in distributing prescriptions, along with providing community–clinical linkages for patients, it is important for prescription programs to offer culturally and linguistically tailored classes and materials to ensure programmatic effectiveness.

Third, minimizing the amount of time required by prescribers to distribute the prescriptions is helpful for effective program implementation. Although streamlining the process of distribution for all implementing partners may not be feasible, one strategy is to move from a paper prescription to a reusable card. Prescribers would issue the card and load it with a certain amount of dollars for fruits and vegetables, and patients would use it just like any other payment card at participating food retailers at point-of-sale. Prescribers could reload cards during follow-up appointments or classes. Although a card-based system may be more expensive than a paper-based system, ultimately it could increase efficiency and improve tracking for distribution and redemption.

Implementing partners worked with various populations, including racial/ethnic minority groups, senior citizens, and residents in urban and rural areas. For all implementing partners, redemption rates were 34% or higher. We realize that a statewide fruit and vegetable prescription program may not be feasible for other states to implement because of lack of funding and resources. However, similar programs at any scope or scale can benefit from the lessons learned in our evaluation. Additionally, such programs can play a role in connecting health care with social determinants, which ultimately can improve population health; therefore, funding organizations and legislators should consider investing in programs that support healthy food purchases for low-income patients.

This evaluation has several limitations. The diverse implementation of the fruit and vegetable prescription program limited the evaluation design, data collection methods, and subsequently the generalizability of findings. WA DOH supported flexibility in program implementation, which increased the number of patients receiving a prescription; however, this flexibility prevented the clear, concise interpretation and translation of results that is possible under the conditions of a controlled trial. Although a redemption rate of 54% is respectable, 46% of prescriptions were not redeemed. Because of the varied approaches in program implementation, we could not collect information from patients who did not redeem the prescription and better understand reasons for not using it. Data collection was logistically and ethically challenging because of the number of implementing partners and prescribers; for this reason, we collected a minimal amount of patient-level data. Additionally, program implementation hindered the collection of preprogram and postprogram measures, so we could not ascertain causal relationships. Finally, information on perceived benefit was limited to self-report, which is subject to bias. More objective measures, such as biometric measures collected from an EMR, could eliminate potential bias, and will be added to data collection activities in future years, where possible. Although the use of the electronic survey to collect data from patients enhanced data collection and minimized administrative burden, the response rate could be improved, and evaluation staff members will continue to work with implementing partners to improve this rate.

Despite these limitations, we believe that these evaluation activities were effective in providing a snapshot of the fruit and vegetable prescription program in Washington State. By using an electronic survey to collect data from patients and having electronic access to implementing partner reports and point-of-sale data, we streamlined the process of data collection, entry, and analysis. Consistent reporting from implementing partners allowed for continuous program quality improvement and provided an easy outlet for partners to report programmatic facilitators and barriers. We also believe that our findings and our approach, compared with those of a controlled trial, more accurately describe best practices for translating a fruit and vegetable prescription program to US settings that would not be appropriate for a controlled trial.
